# Functional response of *Harmonia axyridis* preying on *Acyrthosiphon pisum* nymphs: the effect of temperature

**DOI:** 10.1038/s41598-021-92954-x

**Published:** 2021-06-30

**Authors:** Yasir Islam, Farhan Mahmood Shah, Xu Rubing, Muhammad Razaq, Miao Yabo, Li Xihong, Xingmiao Zhou

**Affiliations:** 1 Hubei Insect Resources Utilization and Sustainable Pest Management Key Laboratory, College of Plant Science and Technology, Huazhong Agricultural University, Wuhan, 430070 China; 2 Department of Entomology, Faculty of Agricultural Sciences and Technology, Bahauddin Zakariya University, Multan, 60000 Pakistan; 3 Tobacco Research Institute of Hubei Province, Wuhan, 430030 China

**Keywords:** Animal behaviour, Climate-change ecology, Conservation biology, Population dynamics

## Abstract

In the current study, we investigated the functional response of *Harmonia axyridis* adults and larvae foraging on *Acyrthosiphon pisum* nymphs at temperatures between 15 and 35 °C. Logistic regression and Roger’s random predator models were employed to determine the type and parameters of the functional response. *Harmonia axyridis* larvae and adults exhibited Type II functional responses to *A. pisum*, and warming increased both the predation activity and host aphid control mortality. Female and 4th instar *H. axyridis* consumed the most aphids. For fourth instar larvae and female *H. axyridis* adults, the successful attack rates were 0.23 ± 0.014 h^−1^ and 0.25 ± 0.015 h^−1^; the handling times were 0.13 ± 0.005 h and 0.16 ± 0.004 h; and the estimated maximum predation rates were 181.28 ± 14.54 and 153.85 ± 4.06, respectively. These findings accentuate the high performance of 4th instar and female *H. axyridis* and the role of temperature in their efficiency. Further, we discussed such temperature-driven shifts in predation and prey mortality concerning prey-predator foraging interactions towards biological control.

## Introduction

The use of integrated pest management strategies based on biocontrol agents has received increasing prominence worldwide^1^. It has been implemented with tremendous success in both fields and greenhouses^2^, in particular with the intent of reducing the large-scale use of pesticides^3^. While the adoption of biological control is desirable, the successful implementation depends upon the comprehensive understanding of predator–prey interactions, owing to their fundamental role towards ecosystem functionality and food web stability^4^. Several methods can be applied to quantifying these interactions^5,6^, including functional response^7^, numerical response^8^, kill rate^9^, and consumption rate^10^. Functional responses describe how the predation rate changes with resource density^11^. Three types of functional responses are typically expected, with consumption rate being linear up to a constant plateau (Type I), parabolic (Type II), or sigmoid (Type III)^12^, depending on whether the parameters of functional response, i.e., the enemy attack rate and prey handling time, vary with prey density. In the type II functional response, the attack rate defines the steepness of the increase in predation with the increase of prey density, and handling time sets the satiation threshold^13^. Natural enemies with high attack rate and low handling time are thought to be the most efficient biocontrol agents^13^. Many sources are known to regulate these functional response parameters^14^.

Temperature is a chief driver of biological systems through the temperature-dependent nature of biological rates (e.g., metabolic rates)^15^. The effect of temperature on biological rates is likely to be realized from physiology up to species level, influencing population growth rates and carrying capacities^16^ as well as ecosystem functions. Temperature can affect prey-predator foraging interactions by altering their behavioural or physiological responses^17^. Warming is shown to increase the predator rate of prey consumption by boosting predator’s metabolic rates^18^. In order to consume a large amount of prey, a predator should be adept at searching for and handling its prey, so that it may spend more time searching on consumption events than on prey handling attempts, therefore a change of predatory behaviour or functional response may be expected under warming^19,20^. For instance, the handling time decreased, consumption rate increased, and the type of functional response changed from Type II to Type III for *Podisus maculiventris* (Say) and *Podisus nigrispinus* (Dallas) (Hemiptera: Pentatomidae) preying on *Spodoptera exigua* (Hübner) (Lepidoptera: Noctuidae) when the temperature increased from 18 °C to 27 °C^21^. Similarly, functional response for *Euborellia annulipes* (Lucas) (Dermaptera: Anisolabididae) preying on larvae *Plutella xylostella* (L.) (Lepidoptera: Plutellidae) changed from Type III at low temperature (i.e., 18 °C) to Type II at higher temperature (i.e., 25 °C and 32 °C)^22^. On the other hand, the temperature change did not change the type of functional response for predators, *Scymnus levaillanti* Mulsant, *Adalia bipunctata* L. and *Cycloneda sanguinea* (L.) (Coleoptera: Coccinellidae) preying on *Aphis gossypii* Glover and *Myzus persicae* (Sulzer) (Hemiptera: Aphididae), despite improved prey killing/handling abilities at higher temperatures^19,23^. This suggests differential effects of temperature on prey-predator pairs, probably for species‐specific differences in their sensitivity to temperature and hunting behaviour^24,25^. A large body of literature is directed towards understanding the consequence of warming on food webs and stability^26,27^. As warmer temperature may destabilize some predator–prey systems through increased predator action or enhanced prey mortality^22,28^, valuing the species-specific responses of prey-predator pairs to temperature can enable the better understanding of how temperature affects food webs.

The pea aphid, *Acyrthosiphon pisum* (Harris) (Hemiptera: Aphididae), originally a Palearctic species^29^, has now become a pest of global concern for pulse and legume producers^30^. It has a broad host range, infesting grass pea (*Lathryus sativus* L.), faba bean (*Vicia faba* L.), pea (*Pisum sativum* L.), alfalfa (*Medicago sativa* L.), chickpea (*Cicer arietinum* L.), lentil (*Lens culinaris* Medik.), and lupin (*Lupinus albus* L.) (Fabales: Fabaceae)^31^ (see Holman^32^ for more data on *A. pisum* host plants). The aphid inflicts injury either directly, i.e., by removing sap from succulent phloem tissues or via injecting phytotoxic saliva, or indirectly, by vectoring multiple plant viruses (e.g., the cucumber mosaic virus, the pea enation mosaic virus, the bean leaf roll virus, and the beet yellow virus) or by producing honeydew, inviting sooty-moulds, subsequently disturbing plant’s photosynthetic and respirational functions^33^. Prolonged infestation by pea aphids can lead to plant stunting, deformation, and discoloration, ultimately reducing crop yields by 35.7%^34^. The broad host range, complex life cycle, and quick adaptation to new environments make it difficult to control this aphid. Moreover, this aphid may develop insecticide resistance, making its control through these means challenging^35^.

Many aphidophagous ladybird beetles (Coleoptera: Coccinellidae) are known to be exploited for conservative or augmentative release biocontrol programs of several economically important aphids in diverse crops, outdoors and in greenhouses^2^, suppressing aphid infestations below economically damaging levels^36^. *Harmonia axyridis* (Pallas) (Coleoptera: Coccinellidae) is a generalist predator, is geographically wide-spread^37^, and has been extensively employed as a biocontrol agent of soft-bodied insects, including aphids in a diversity of crops^38^. Various biological aspects of *H. axyridis* relevant to its predatory potential (e.g., phenological characteristics, life table parameters, and generally Type II functional response), have been investigated with respect to temperature and other factors^39,40^. Although functional responses have been measured for *H. axyridis* on many crop pests^41,42^, information on the predation and functional response of *H. axyridis* to *A. pisum* is limited^43^. As the foraging behaviour of coccinellids can differ across predator species, developmental growth stages^41,44^, and different types of prey^45^, the quantification of species-specific prey-predator interactions can improve our understanding of foraging interactions and support the development of efficient biological control.

Here, we report the functional response of *H. axyridis* to *A. pisum* under a range of temperatures. We expect that, based on its close association with predator growth and development, the temperature change also will modify consumption. Further, we aim to assess whether thermal conditions and aphid density affect the functional response of larvae and adult *H. axyridis*. Alongside these, the density-dependent mortality of prey in response to warming was also assessed.

## Results

**Figure 1:**
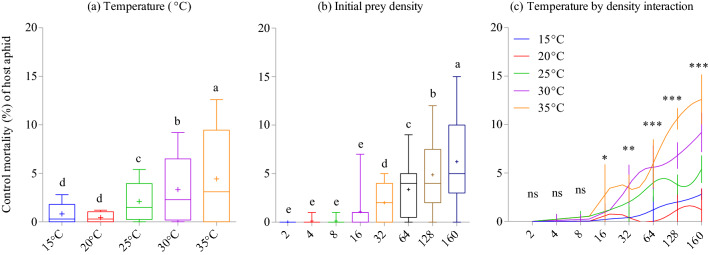
Percent control mortality of *Acyrthosiphon pisum* nymphs exposed to different thermal conditions under different prey densities. Data are expressed with box plots for temperature (**a**), prey density effects (**b**), and through line graphs for density-wise temperature effects (**c**) on mean percent mortalities (with 95% confidence intervals (CIs)) following significant temperature by density interaction. Box plots are showing the range of data (lower and upper quartiles and extreme values), median, and mean (symbols), and different alphabets indicate significant differences between group means (*P* < 0.05; Tukey’s HSD test). In panel c, the temperature effects are tested within each prey density offered through running independent ANOVAs and significant means are compared according to non-overlapping 95% CI of difference. Symbols ‘*’, ‘**’, and ‘***’ denote significance at 0.05, 0.01, and 0.001 levels, respectively, whereas ‘ns’ indicates non-significant (*P* > 0.05) differences for ANOVAs performed.

Aphid mortality increased with increasing host aphid density and temperature (temperature: *F*_4,160_ = 78.64; *P* < 0.001; Fig. 1a; density: *F*_7,160_ = 116.75; *P* < 0.001; Fig. 1b). The density-wise assessment showed similar behaviour for increasing mortality with respect to warming and increasing aphid densities (*F*_28,160_ = 10.23; *P* < 0.001; Fig. 1c).

**Figure 2:**
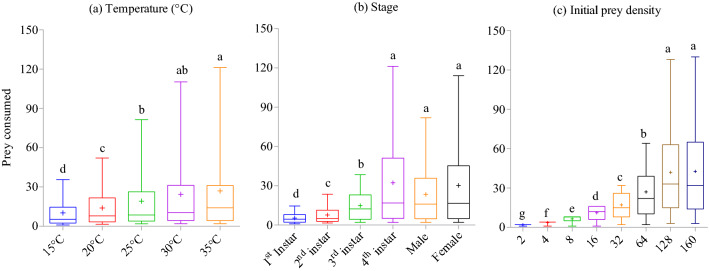
Mean number of *Acyrthosiphon pisum* nymphs consumed by *Harmonia axyridis* under different thermal conditions (**a**), growth stages (**b**), and densities of prey offered (**c**). Box plots are showing the range of data (lower and upper quartiles, and extreme values), median, and mean (symbols), and different alphabets indicate significant differences among group means according to Wald chi square test with 95% CI of difference.

**Figure 3:**
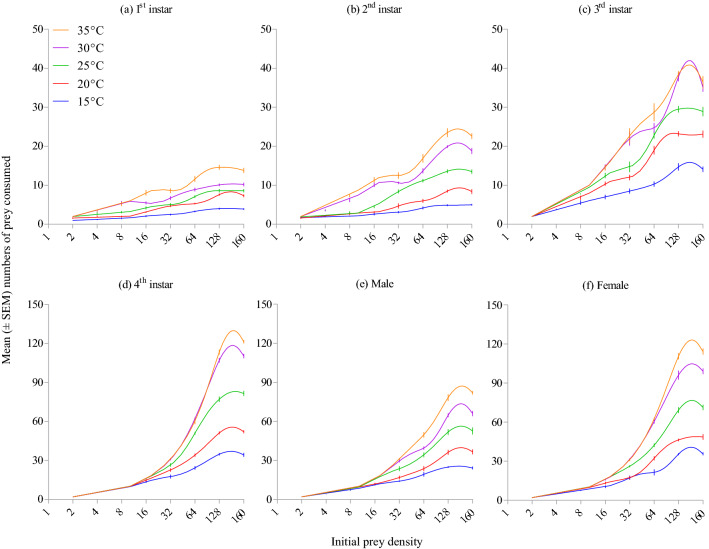
Mean (± SEM) numbers of *Acyrthosiphon pisum* nymphs consumed by *Harmonia axyridis* under different thermal conditions at different prey densities.

**Figure 4:**
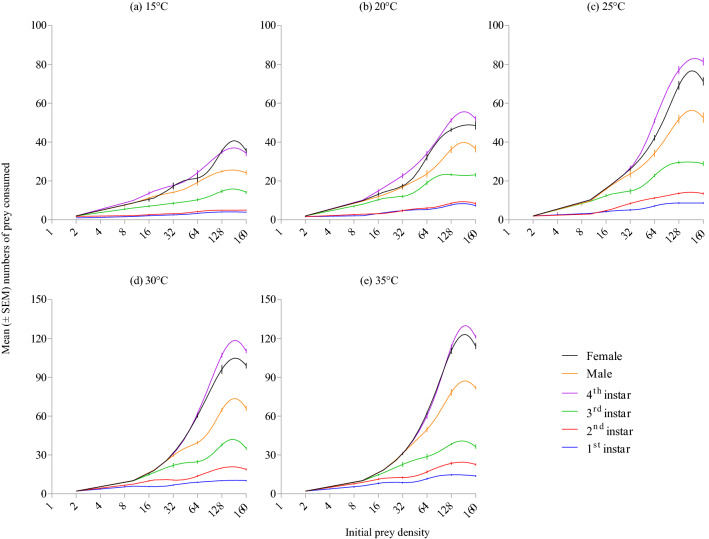
Mean (± SEM) numbers of *Acyrthosiphon pisum* nymphs consumed by various growth stages of *Harmonia axyridis* under different thermal conditions at different prey densities.

*Harmonia axyridis* rate of prey consumption increased with warmer temperatures (Wald *X*^2^ = 104.86; df = 4; *P* < 0.001; Fig. 2a), with consumption significantly greater at 30 °C (i.e., 24.41 ± 6.11 aphids/day) and 35 °C (i.e., 27.03 ± 6.43 aphids/day) than at colder temperatures. The rate of prey consumption was significantly different between growth stages of the predator (Wald *X*^2^ = 539.39; df = 5; *P* < 0.001; Fig. 2b) and densities of prey offered (Wald *X*^2^ = 1759.52; df = 7; *P* < 0.001; Fig. 2c). Adults (female: 30.21 ± 5.12 aphids/day; male: 23.45 ± 3.71 aphids/day) and 4th instar (32.25 ± 5.27 aphids/day) of this predator consumed significantly more aphids than did younger instars. Prey consumption was the highest at prey densities of 128 and 160 aphids/petri dish arena. Three way (temperature × stage × density: Wald *X*^2^ = 15.07; df = 140; *P* = 1) and two way (temperature × stage: Wald *X*^2^ = 27.78; df = 20; *P* = 0.115; temperature × density: Wald *X*^2^ = 20.24; df = 28; *P* = 0.855) interactions were non-significant, meaning the temperature (Fig. 3a-f) and growth stage (Fig. 4a-e) effects on mean prey consumption remained unchanged when effects were assessed at each prey density offered.

**Figure 5:**
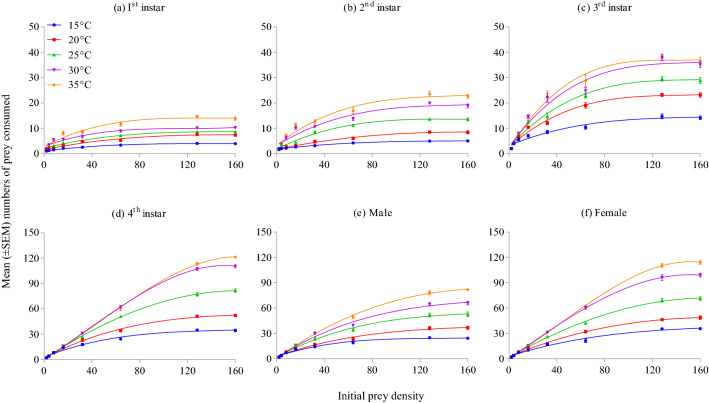
Type II functional response curves fitted by Roger’s random predator equation of *Harmonia axyridis* under different densities of *Acyrthosiphon pisum* over a 24 h period, under different thermal conditions.

**Figure 6:**
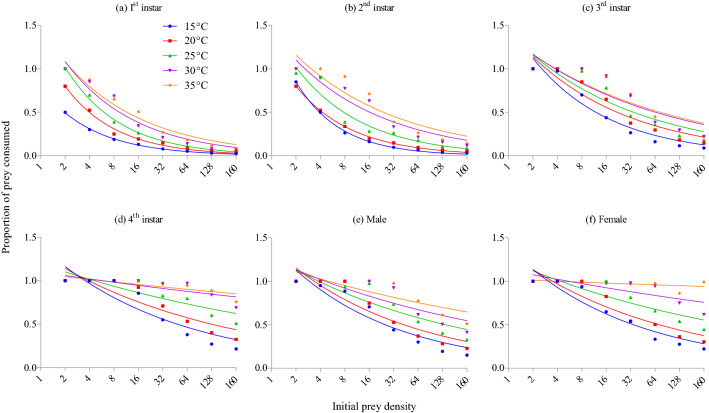
Proportion of *Acyrthosiphon pisum* nymphs consumed by *Harmonia axyridis* under different thermal conditions at different prey densities.

Logistic regression between the initial aphid densities offered and the proportion of aphid consumed (*Na*/*No*) showed all significantly negative values of the linear coefficients ${P}_{1}$; indicating a Type II functional response across all growth stages and temperatures tested (Table 1). The declining consumption with increasing aphid densities (Fig. 5) also confirmed a Type II functional response. The monotonically declining proportion of consumption with increased aphid densities, led to further confirmation of Type II functional responses (Fig. 6). A more linear decline for the proportion of prey eaten with increasing aphid density was noted for 4th instar (Fig. 6d) and female *H. axyridis* (Fig. 6f) at higher (i.e., 30 and 35 °C) than lower temperatures.

**Table 1:** Results of the logistic regression analysis of the proportion of nymphs of *Acyrthosiphon pisum* predated by all stages of *Harmonia axyridis* relative to the initial number of nymphs provided.

Temperatures	Growth Stages	Parameters	Estimates	S.E	*Z*-Value	*Pr* (z)
15 °C	1st instar	Intercept	− 7.39 × 10^−01^	2.26 × 10^−01^	− 3.26	0.001
		Linear	− 6.96 × 10^−02^	1.37 × 10^−02^	− 5.05	4.36 × 10^−07^
		Quadratic	6.449 × 10^−04^	1.962 × 10^−04^	3.286	1.01 × 10^−03^
		Cubic	− 2.040 × 10^−06^	7.740 × 10^−07^	− 2.635	8.40 × 10^−03^
	2nd instar	Intercept	− 3.67 × 10^−02^	2.03 × 10^−01^	− 0.18	0.85
		Linear	− 8.96 × 10^−02^	1.25 × 10^−02^	− 7.15	8.15 × 10^−13^
		Quadratic	8.635 × 10^−04^	1.771 × 10^−04^	4.876	1.08 × 10^−06^
		Cubic	− 2.73 × 10^−06^	6.95 × 10^−07^	− 3.941	8.12 × 10^−05^
	3rd instar	Intercept	1.58	1.93 × 10^−01^	8.19	2.42 × 10^−16^
		Linear	− 1.06 × 10^−01^	9.99 × 10^−03^	− 10.67	< 2 × 10^−16^
		Quadratic	1.03 × 10^−03^	1.30 × 10^−04^	7.887	3.09 × 10^−15^
		Cubic	− 3.24 × 10^−06^	4.89 × 10^−07^	− 6.624	3.50 × 10^−11^
	4th instar	Intercept	3.35	2.62 × 10^−01^	12.76	< 2 × 10^−16^
		Linear	− 1.18 × 10^−01^	1.08 × 10^−02^	− 10.93	< 2 × 10^−16^
		Quadratic	1.08 × 10^−03^	1.26 × 10^−04^	8.580	< 2 × 10^−16^
		Cubic	− 3.30 × 10^−06^	4.43 × 10^−07^	− 7.462	8.52 × 10^−14^
	Male	Intercept	2.30	2.13 × 10^−01^	10.77	< 2 × 10^−16^
		Linear	− 9.59 × 10^−02^	9.68 × 10^−03^	− 9.91	< 2 × 10^−16^
		Quadratic	8.61 × 10^−04^	1.20 × 10^−04^	7.176	7.20 × 10^−13^
		Cubic	− 2.62 × 10^−06^	4.35 × 10^−07^	− 6.012	1.84 × 10^−09^
	Female	Intercept	2.47	2.19 × 10^−01^	11.27	< 2 × 10^−16^
		Linear	− 9.75 × 10^−02^	9.60 × 10^−03^	− 10.15	< 2 × 10^−16^
		Quadratic	9.13 × 10^−04^	1.16 × 10^−04^	7.877	3.36 × 10^−15^
		Cubic	− 2.81 × 10^−06^	4.13 × 10^−07^	− 6.808	9.90 × 10^−12^
20 °C	1st instar	Intercept	− 8.50 × 10^−02^	1.95 × 10^−01^	− 0.43	0.66
		Linear	− 7.39 × 10^−02^	1.12 × 10^−02^	− 6.55	5.42 × 10^−11^
		Quadratic	7.037 × 10^−04^	1.561 × 10^−04^	4.509	6.52 × 10^−06^
		Cubic	− 2.237 × 10^−06^	6.045 × 10^−07^	− 3.700	2.16 × 10^−04^
	2nd instar	Intercept	2.97 × 10^−02^	1.92 × 10^−01^	0.155	0.87
		Linear	− 7.59 × 10^−02^	1.09 × 10^−02^	− 6.92	4.32 × 10^−12^
		Quadratic	7.33 × 10^−04^	1.50 × 10^−04^	4.871	1.11 × 10^−06^
		Cubic	− 2.33 × 10^−06^	5.79 × 10^−07^	− 4.031	5.56 × 10^−05^
	3rd instar	Intercept	2.01	2.03 × 10^−01^	9.92	< 2 × 10^−16^
		Linear	− 9.10 × 10^−02^	9.43 × 10^−03^	− 9.64	< 2 × 10^−16^
		Quadratic	8.24 × 10^−04^	1.18 × 10^−04^	6.959	3.43 × 10^−12^
		Cubic	− 2.53 × 10^−06^	4.33 × 10^−07^	− 5.838	5.29 × 10^−09^
	4th instar	Intercept	3.82	3.08 × 10^−01^	12.41	< 2 × 10^−16^
		Linear	− 1.09 × 10^−01^	1.18 × 10^−02^	− 9.25	< 2 × 10^−16^
		Quadratic	9.71 × 10^−04^	1.32 × 10^−04^	7.337	2.19 × 10^−13^
		Cubic	− 2.90 × 10^−06^	4.48 × 10^−07^	− 6.471	9.74 × 10^−11^
	Male	Intercept	2.85	2.36 × 10^−01^	12.04	< 2 × 10^−16^
		Linear	− 1.06 × 10^−01^	1.00 × 10^−02^	− 10.51	< 2 × 10^−16^
		Quadratic	9.86 × 10^−04^	1.19 × 10^−04^	8.232	< 2 × 10^−16^
		Cubic	− 3.02 × 10^−06^	4.22 × 10^−07^	− 7.152	8.54 × 10^−13^
	Female	Intercept	2.68	2.36 × 10^−01^	11.39	< 2 × 10^−16^
		Linear	− 8.48 × 10^−02^	9.81 × 10^−03^	− 8.64	< 2 × 10^−16^
		Quadratic	7.68 × 10^−04^	1.15 × 10^−04^	6.661	2.72 × 10^−11^
		Cubic	− 2.35 × 10^−06^	4.04 × 10^−07^	− 5.827	5.65 × 10^−09^
25 °C	1st instar	Intercept	4.89 × 10^−01^	1.87 × 10^−01^	2.61	0.009
		Linear	− 8.64 × 10^−02^	1.06 × 10^−02^	− 8.12	4.54 × 10^−16^
		Quadratic	8.267 × 10^−04^	1.463 × 10^−04^	5.652	1.59 × 10^−08^
		Cubic	− 2.619 × 10^−06^	5.645 × 10^−07^	− 4.639	3.51 × 10^−06^
	2nd instar	Intercept	4.75 × 10^−01^	1.78 × 10^−01^	2.65	0.007
		Linear	− 6.27 × 10^−02^	9.40 × 10^−03^	− 6.67	2.48 × 10^−11^
		Quadratic	5.60 × 10^−04^	1.26 × 10^−04^	4.435	9.22 × 10^−06^
		Cubic	− 1.75 × 10^−06^	4.81 × 10^−07^	− 3.641	2.71 × 10^−04^
	3rd instar	Intercept	2.73	2.31 × 10^−01^	11.79	< 2 × 10^−16^
		Linear	− 1.04 × 10^−01^	1.00 × 10^−02^	− 10.38	< 2 × 10^−16^
		Quadratic	9.53 × 10^−04^	1.21 × 10^−04^	7.859	3.86 × 10^−15^
		Cubic	− 2.92 × 10^−06^	4.32 × 10^−07^	− 6.749	1.49 × 10^−11^
	4th instar	Intercept	4.12	4.02 × 10^−01^	10.25	< 2e − 16
		Linear	− 7.67 × 10^−02^	1.47 × 10^−02^	− 5.21	1.89 × 10^−07^
		Quadratic	5.94 × 10^−04^	1.58 × 10^−04^	3.755	1.73 × 10^−04^
		Cubic	− 1.71 × 10^−06^	5.18 × 10^−07^	− 3.312	9.26 × 10^−04^
	Male	Intercept	4.03	3.24 × 10^−01^	12.41	< 2 × 10^−16^
		Linear	− 1.13 × 10^−01^	1.23 × 10^−02^	− 9.22	< 2 × 10^−16^
		Quadratic	9.95 × 10^−04^	1.36 × 10^−04^	7.307	2.73 × 10^−13^
		Cubic	− 2.94 × 10^−06^	4.58 × 10^−07^	− 6.429	1.29 × 10^−10^
	Female	Intercept	4.95	4.22 × 10^−01^	11.73	< 2 × 10^−16^
		Linear	− 1.28 × 10^−01^	1.50 × 10^−02^	− 8.52	< 2 × 10^−16^
		Quadratic	1.15 × 10^−03^	1.59 × 10^−04^	7.259	3.89 × 10^−13^
		Cubic	− 3.48 × 10^−06^	5.18 × 10^−07^	− 6.718	1.84 × 10^−11^
30 °C	1st instar	Intercept	1.22	1.90 × 10^−01^	6.46	1.04 × 10^−10^
		Linear	− 1.00 × 10^−01^	1.03 × 10^−02^	− 9.75	< 2 × 10^−16^
		Quadratic	9.454 × 10^−04^	1.394 × 10^−04^	6.781	1.19 × 10^−11^
		Cubic	− 2.942 × 10^−06^	5.334 × 10^−07^	− 5.515	3.48 × 10^−08^
	2nd instar	Intercept	1.98	2.02 × 10^−01^	9.82	< 2 × 10^−16^
		Linear	− 1.05 × 10^−01^	9.75 × 10^−03^	− 10.77	< 2 × 10^−16^
		Quadratic	9.99 × 10^−04^	1.24 × 10^−04^	8.060	7.61 × 10^−16^
		Cubic	− 3.12 × 10^−06^	4.56 × 10^−07^	− 6.833	8.29 × 10^−12^
	3rd instar	Intercept	4.40	3.25 × 10^−01^	13.52	< 2 × 10^−16^
		Linear	− 1.46 × 10^−01^	1.26 × 10^−02^	− 11.58	< 2 × 10^−16^
		Quadratic	1.34 × 10^−03^	1.41 × 10^−04^	9.465	< 2 × 10^−16^
		Cubic	− 4.06 × 10^−06^	4.82 × 10^−07^	− 8.420	< 2 × 10^−16^
	4th instar	Intercept	4.97	7.80 × 10^−01^	6.37	1.87 × 10^−10^
		Linear	− 3.00 × 10^−02^	2.86 × 10^−02^	− 1.04	0.29
		Quadratic	6.043e − 05	3.021e − 04	0.200	0.841
		Cubic	− 2.243e − 07	9.595e − 07	− 0.234	0.815
	Male	Intercept	7.65	6.89 × 10^−01^	11.09	< 2 × 10^−16^
		Linear	− 2.10 × 10^−01^	2.25 × 10^−02^	− 9.32	< 2 × 10^−16^
		Quadratic	1.87 × 10^−03^	2.22 × 10^−04^	8.433	< 2 × 10^−16^
		Cubic	− 5.47 × 10^−06^	6.86 × 10^−07^	− 7.986	1.4 × 10^−15^
	Female	Intercept	4.96	7.32 × 10^−01^	6.77	1.25 × 10^−11^
		Linear	− 3.59 × 10^−02^	2.60 × 10^−02^	− 1.38	0.16
		Quadratic	2.75 × 10^−05^	2.69 × 10^−04^	0.102	0.919
		Cubic	1.38 × 10^−07^	8.49 × 10^−07^	0.163	0.871
35 °C	1st instar	Intercept	1.42	1.89 × 10^−01^	7.50	6.00 × 10^−14^
		Linear	− 9.36 × 10^−02^	9.71 × 10^−03^	− 9.63	< 2 × 10^−16^
		Quadratic	8.732 × 10^−04^	1.280 × 10^−04^	6.823	8.92 × 10^−12^
		Cubic	− 2.731 × 10^−06^	4.828 × 10^−07^	− 5.658	1.54 × 10^−08^
	2nd instar	Intercept	2.57	2.22 × 10^−01^	11.57	< 2 × 10^−16^
		Linear	− 1.15 × 10^−01^	1.01 × 10^−02^	− 11.37	< 2 × 10^−16^
		Quadratic	1.089 × 10^−03^	1.25 × 10^−04^	8.706	< 2 × 10^−16^
		Cubic	− 3.37 × 10^−06^	4.52 × 10^−07^	− 7.458	8.77 × 10^−14^
	3rd instar	Intercept	4.00	3.12 × 10^−01^	12.81	< 2 × 10^−16^
		Linear	− 1.21 × 10^−01^	1.21 × 10^−02^	− 10.03	< 2 × 10^−16^
		Quadratic	1.05 × 10^−03^	1.36 × 10^−04^	7.714	1.22 × 10^−14^
		Cubic	− 3.11 × 10^−06^	4.66 × 10^−07^	− 6.672	2.52 × 10^−11^
	4th instar	Intercept	6.90	1.07	6.44	1.13 × 10^−10^
		Linear	− 1.22 × 10^−01^	3.59 × 10^−02^	− 3.40	0.0006
		Quadratic	1.14 × 10^−03^	3.59 × 10^−04^	3.173	1.50 × 10^−04^
		Cubic	− 3.75 × 10^−06^	1.11 × 10^−06^	− 3.385	7.13 × 10^−04^
	Male	Intercept	9.20	1.15	7.94	1.94 × 10^−15^
		Linear	− 2.25 × 10^−01^	3.61 × 10^−02^	− 6.25	4.08 × 10^−10^
		Quadratic	1.93 × 10^−03^	3.41 × 10^−04^	5.660	1.51 × 10^−08^
		Cubic	− 5.50 × 10^−06^	1.01 × 10^−06^	− 5.437	5.43 × 10^−08^
	Female	Intercept	5.13	8.52 × 10^−01^	6.02	1.72 × 10^−09^
		Linear	− 2.91 × 10^−02^	3.14 × 10^−02^	− 0.92	0.35
		Quadratic	6.93 × 10^−05^	3.31 × 10^−04^	0.209	0.834
		Cubic	− 3.27 × 10^−07^	1.05 × 10^−06^	− 0.311	0.756

**Figure 7:**
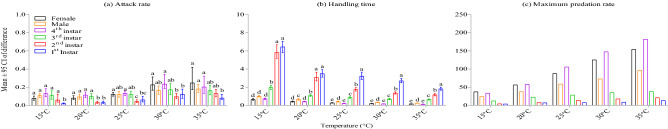
Functional response parameters of *Harmonia axyridis* preying on *Acyrthosiphon pisum* nymphs under different thermal conditions, resulting from bootstrapped functional response parameters. Different letters above the bars within each temperature indicate significant difference (*P* < 0.05) among growth stages of the predator based upon non-overlapping 95% CI of difference.

Estimates of functional response parameters, determined through fits to the Rogers random predator model, revealed that the *H. axyridis* exhibited the highest attack rate (*a*) (Fig. 7a), the shortest handling time ${(T}_{h})$ (Fig. 7b), and the maximum predation rate (*T*/*Th*) (Fig. 7c) typically at higher temperatures and later growth stages. The attack rates (*a*) and maximum predation rates (*T*/*Th*) were generally low at low temperatures (15 and 20 °C) that started to increase with warming, reaching a peak at 30 and 35 °C. On the other hand, the handling time ${(T}_{h})$ was the lowest at 35 °C and increased with lower temperatures. The estimate of maximum predation rate (*T*/*Th*) was the highest for 4th instar *H. axyridis* followed by the female and male *H. axyridis*, respectively. The 4th instar and female *H. axyridis* similarly showed the lower handling times ${(T}_{h})$ than male *H. axyridis*.

## Discussion

Insect ectotherms are known to respond to thermal conditions for their development, biology, and population dynamics^46,47^, as well as through their trophic interactions^48,49^. The current research explored the functional response of *H. axyridis* foraging on *A. pisum* at different growth stages, temperatures and evaluated the effect of temperature on host aphid mortality. The temperature ranges we tested are relevant across a range of temperate or sub-tropical regions. Our results showed increasing host aphid mortality with warmer temperatures, meaning warming could lead to faster prey depletion. A low-temperature threshold (i.e., 20 °C) is reported the best for aphid growth and development, whereas temperatures > 30 °C have been shown to become unfavourable for aphid fertility and development, subsequently compromising population buildup^50^. We showed a Type II functional response by all stages of *H. axyridis*, within the tested host aphid density ranges and thermal conditions, with maximum predation by the 4th instar and female *H. axyridis* between 25 and 35 °C. Warming also has been shown to accelerate the pace of insect metamorphosis. Accelerated growth/development enhances metabolic rate and energy gain requirements^41,51^, which predators may meet by consuming large meals^24^, possibly explaining our results of heightened predation under warming. Heightened predation under warming (between 14 and 35 °C) has been observed for *H. axyridis* preying on other species of aphids (*Chromaphis juglandicola* Kalt. or *Panaphis juglandis *(Goeze))^40^ as well as eggs of *Spodoptera litura* F. (Lepidoptera: Noctuidae)^41^, and for other coccinellids, including *A. bipunctata*, *Hippodamia convergens* Guérin-Méneville and *Coccinella septempunctata* L., preying on *M. persicae*^19,52^, suggesting some generality of this outcome.

In our findings, despite its positive effect on aphid consumption, changing temperature did not change *H. axyridis* functional response type (i.e., from Type II to Type III). A changing functional response type with thermal changes has been reported for many predators^22^, but rarely for coccinellids. A Type II response describes an increase in predation with increasing prey abundance, gradually decelerating to an asymptotic foraging rate at higher abundance^7^. This also generates negative density-dependent mortality, often a characteristic of predators that provide efficient control at smaller resource^53^. Among the three types of functional responses described by Holling^7^, only type producing a positive density dependent mortality is Type III^11^. The only possibility for exhibiting a Type III response in current study is the concentration of predator hunting in high density patches^54^. This mechanism could have operated in the current experiments, however, no evidence of Type III response was found in our data. The other mechanisms for a Type III response include switching behaviour and predator learning that could not have operated in our system, as the experiments were short-term and single prey-based. Furthermore, *H axyridis* often shows a Type II functional response. Type II functional responses have been found for *H. axyridis* when preying on immature *S. litura*^41^, or when preying on *C. juglandicola* or *P. juglandis* at different temperatures^40^, *Rhopalosiphum padi* L. and *Sitobion avenae* F. with different fertilizer treatments^55^, or different prey types such as *Lipaphis erysimi* K. (Hemiptera: Aphididae), *Cacopsylla chinensis* (Hemiptera: Psyllidae) and *Danaus plexippus* L. (Lepidoptera: Nymphlidae)^42,56,57^, or different growth stages of *A. gossypii*, *M. persicae*, *Myzus nicotianae* S., *Aphis glycines* Matsumura (Hemiptera: Aphididae) and *Diaphorina citri* (Hemiptera: Psyllidae)^58–61^. However, the change of functional response type with respect to prey distribution^62^ or prey quality (either pesticide treated or untreated) have been shown for *H. axyridis*^63,64^, suggesting complex nature of predator–prey interactions, and, emphasizing the need for further assessments of the functional response with regard to factors like pesticides^33^. Insecticides from synthetics and biopesticides groups are commonly applied worldwide^65–68^ and have been shown to have profound effects, both positive or negative, on behavioural or physiological responses of predators^39,69^.

The attack rate and handling time describe the overall functional response magnitude. The attack rate (also called the space clearance rate or attack efficiency) describes the ability a predator possesses to catch its prey in a given time frame, and handling time describes the time lost from searching per host consumed^70^. A high attack rate means that the predator is adept at quickly removing hosts from the volumes or areas it is searching, and low handling time means how quickly a predator traps, hunts, and digests prey^70^. In our findings, the parameter estimates showed greater variation across predator growth stages, with frequently higher estimates at later growth stages and higher temperatures. Maximum daily predation rates were temperature-dependent, especially their increase, and the handling time of *H. axyridis* showed an exponential decrease for all growth stages at the lowest thermal conditions, meaning predators will spend less investment on foraging, possibly using that time for resting and decrease predation^71,72^. Conversely, the greater number of prey consumed due to warming may have resulted from a decrease in handling time with a resultant increase in encounter rates^73^. The expected increase in metabolic rates with warming are associated with greater energy demands, which should cause predators to increase food intake and foraging activity. Determining these parameters with respect to phenological stage confirmed poor response by the first three instars as reported earlier^59,74^, whereas increased foraging performance by final instar and adult *H. axyridis* (especially female when compared with male)^24,45,52^ suggesting *H. axyridis* may provide better biocontrol efficiency at later stages, plausibly owing to better searching efficiencies. The 4th instar requires large meals to attain the required weight for pupation^75^ and adult predators have to prepare for reproduction^56^ and other functions related to egg maturation or fertilization^76^. Our results of greater predation by the 4th instar and female *H. axyridis* are supported by previous reports investigating the functional response of coccinellids, namely *Adalia tetraspilota* (Hope), *Hippodamia variegata* (Goeze), *Harmonia dimidiata* (Fab.), and *Scymnus syriacus* Marseul preying on many aphid species^44,45,77^.

Our results showed strong potential of *H. axyridis* for *A. pisum* control. The 4th instar and female *H. axyridis* emerged as the best performing biocontrol candidates. We determined the strong role of temperature in predator’s efficiency, as accelerated predator action under warming (30 and 35 °C) increased prey consumption. However, there was no evidence for functional response type change with warmer temperatures. Another striking result was that prey mortality increased with warming. This indicates that warming may reduce prey availability by increasing predator action and enhancing prey mortality, which allows us to suggest that *H. axyridis* release at warmer temperatures may not be feasible against this aphid pest. The 4th instar and female *H. axyridis* can be best used at low temperatures (between 20 and 25 °C) against this aphid pest. Prey depletion under warming can affect predator–prey interactions by modifying functional responses that can then destabilize communities through intraguild predation or other antagonistic interactions triggered in response to prey depletion^28,78^. This necessitates the need for further studies exploring alternative prey resources to support this generalist predator in the management of aphid pests under warmer conditions.

## Methods

Cucumber (*Cucumis sativa* L., cv. Negin; Cucurbitales: Cucurbitaceae) and broad bean (*Vicia faba* L.; Fabales: Fabaceae) seedlings were grown from seeds purchased from Caoxian County, Shandong, China. The seeds were sown in pots (25 cm diameter, 30 cm deep) with (3:1) soil: manure. The seedlings were maintained under greenhouse conditions of 12–26 °C, 45–55% RH, and 16:8 h (Light: Dark) photoperiod, and subsequently used for rearing and conducting functional response assays. The plant materials used were obtained with prior permission, and the present study is in compliance with relevant guidelines and legislation.

For establishing *A. pisum* culture, the initial populations of aphid collected from unsprayed alfalfa fields were subsequently brought to the laboratory and reared on broad bean plants inside net cages (20 × 10 × 30 cm height). The stock culture of *H. axyridis* was developed from a pre-established laboratory colony, already available in the same laboratory. The predator was reared on *A. pisum* infested bean plants (7–8 leaves) inside net cages (60 × 42 × 30 cm height) for three consecutive generations at laboratory conditions of 24 ± 1 °C, 65 ± 5% RH and 16:8 h (Light: Dark) photoperiod. Bean plants were checked daily for predator eggs. When found, egg batches were carefully removed, placed on tissue paper in Petri dishes (9 cm), and transferred to a computer-operated growth chamber, maintained at settings of 25 ± 1 °C, 65 ± 5% RH and 16:8 h (Light: Dark) photoperiod. The post-emergence larvae were separated and reared in Petri dishes containing aphids as their diet, refreshed daily. The whole culture was maintained at the Department of Plant Protection, Huazhong Agricultural University, China.

The experimental arena consisted of clear Petri dishes (9 cm diameter), with a micromesh screen over the top for ventilation and bottom covered with clean cucumber leaf disk. The desiccation of cucumber leaf disc was prevented by adding 1% agar solution^79^. The assays were performed with *H. axyridis* larvae (i.e., 1st instar, 2nd instar, 3rd instar, 4th instar) and adults (male, female) at five constant temperatures (i.e., 15, 20, 25, 30, 35 °C). The homogeneity of predator age was maintained within each tested growth stage. The first instar larvae were separated one by one shortly after hatching to avoid sibling cannibalism. Hatchlings were reared in Petri dishes (9 cm diameter) until maturity on 4th instar nymphs (100–150 aphids/day). Female *H. axyridis* included mated individuals^59^. First instar larvae were starved for about 6 h, whereas subsequent instars/stages were starved for 24 h to standardize hunger level, according to Islam, et al.^41^. The moist cotton roll offered humidity to all predators during starvation. The use of 4th instar aphid was ensured throughout the experiments as a way to prevent predator preference switch according to prey size^80^. Using a fine camel hairbrush, the aphids at different densities (i.e., 2, 4, 8, 16, 32, 64, 128, and 160 aphids) were transferred in Petri dishes, allowed to spread and settle over the substrate for 30 min, and thereafter were transferred to a computerized growth chamber at the experimental temperatures (i.e., 15, 20, 25, 30, 35 °C), with 70 ± 5% RH and a 16:8 h (Light: Dark) photoperiod. The whole experiment was replicated 10 times for each prey density, growth stage, and temperature combination. The numbers of aphid consumed were recorded every 24 h. Five control replicates were performed for assessing the prey mortality concerning thermal conditions imposed and prey densities offered. Control replicates were kept free from *H. axyridis* to account for natural mortality and to correct for *A. pisum* consumption by the predator as a function of natural mortality. Predation mortality data were corrected for control mortality by applying Abbott's correction^81^.

The control mortality data were analyzed between temperatures, aphid densities, and their interaction, by using Univariate Analysis of Variance (ANOVA) in SPSS (version 21), fitting the above three variables as fixed factors against the dependent variable (i.e., host mortality). Significant (*P* < 0.05) effects were further compared by using Tukey’s Honestly Significant Difference (HSD) multiple comparisons test. Prior to analysis, the mortality data were tested for normality and homogeneity of error variance (i.e., homoscedasticity) using Shapiro–Wilk and Levene tests, and Y = √ x + 1 transformed to improve compliance with these assumptions. All means and standard errors in text and figures are calculated with untransformed data.

Aphid consumption by *H. axyridis* for temperature, growth stage, density, and their two-way and three-way interactions were analyzed by using Generalized Linear Models (GLM) in SPSS (version 21). Kolmogorov–Smirnov test confirmed non-normal distributions of data (*P* > 0.05), and due to over-dispersion, the data were fitted with negative binomial distribution and a log link function, and factors and interaction effects were analyzed by using the Wald Chi-Square test for a confidence level (CI) of 95%. If needed, the multiple follow up tests (GLM) were run to analyze the temperature and growth stage effects separately across prey densities offered, using SPSS (version 21), and the significance for each test was adjusted by following Bonferroni correction (i.e., dividing the standard *P*-value criterion by the number of tests) to avoid Type 1 error.

Analysis of the functional response was done in two different phases^11^, in the R statistical environment^82^. The first phase involved the determination of type and estimation of the parameters of the functional response curve. It is compulsory to find the type of functional response for calculating the functional response parameters using a proper model. The type was determined by applying logistic regression of the proportion of prey eaten as a function of initial prey density offered. A polynomial logistic regression equation assuming a binomial distribution of data to define the type of functional response^11^ (Eq. 1) was fitted as under:

1 $$\frac{{N_{a} }}{{N_{0} }} = \frac{{\exp \left( {P_{o} + P_{1} N_{o} + P_{2} N_{o}^{2} + P_{3} N_{o}^{3} } \right)}}{{1 + \exp \left( {P_{o} + P_{1} N_{o} + P_{2} N_{o}^{2} + P_{3} N_{o}^{3} } \right)}}$$

where *N*_*a*_ and *N*_*o*_ indicate the number of prey consumed and the initial prey density offered, respectively, and $\frac{Na}{No}$ is the proportion of prey consumed. The *P*_o_, *P*_1_, *P*_2_, and *P*_3_ are the regression parameters representing intercept or constant, linear, quadratic, and cubic coefficients, respectively. The coefficients were calculated using maximum likelihood. The values of the linear and quadratic coefficients indicate the nature of functional response, either Type II or Type III. When the value of a linear parameter is negative, the functional response is Type II, and if it is positive with a negative quadratic coefficient, then response is of Type III. The Type II response shows that the proportion of prey consumption decreases as the prey density increases, and a Type III response represents that the proportion of prey consumed increases until an inflection point and then decreases^11^. Once the functional response type was determined, the second phase started where functional response parameters were determined. Data were fitted to the Rogers’ type II random predator equation, using non-linear least square regression, as the prey was not replaced during the entire experiment ^83^. The attack rate (*a*) and handling time (*Th*) were calculated by using the random predator model as under (Eq. 2):

2 $$N_{a} = N_{o} \left[ {1 - \exp \left( {a\left( {T_{h} N_{{a}} - T} \right)} \right)} \right]$$

where ${N}_{a}$ is the number of prey eaten, ${N}_{o}$ is the initial prey density (prey.arena^−1^), $a$ is the attack rate (arena.hour^−1^), ${T}_{h}$ is the handling time (hour.prey^−1^), and *T* is time available for predator during the experiment (here 24 h). Here, the “*glm*” function was used to fit the logistic regression, and the parameters (attack rate $a$ and handling time ${T}_{h}$) of functional response were estimated by using *FRAIR* (Functional Response Analysis in R, version 4.0.0)^84^. The maximum theoretical predation rate per day (K = T/T_h_)^85^ indicates the maximum amount of prey that a predator can consume in a given time frame (here 24 h).
